# Combination of CD8β Depletion and Interleukin-15 Superagonist N-803 Induces Virus Reactivation in Simian-Human Immunodeficiency Virus-Infected, Long-Term ART-Treated Rhesus Macaques

**DOI:** 10.1128/JVI.00755-20

**Published:** 2020-09-15

**Authors:** Julia B. McBrien, Andrew K. H. Wong, Erick White, Diane G. Carnathan, John H. Lee, Jeffrey T. Safrit, Thomas H. Vanderford, Mirko Paiardini, Ann Chahroudi, Guido Silvestri

**Affiliations:** a Emory Vaccine Center and Yerkes National Primate Research Center, Emory University, Atlanta, Georgia, USA; b NantKwest, Culver City, California, USA; c Department of Pediatrics, Emory University, Atlanta, Georgia, USA; Icahn School of Medicine at Mount Sinai

**Keywords:** AIDS, depletion, HIV, IL-15, immunology, latency, N-803, reservoir, SHIV, SIV

## Abstract

The “shock and kill” strategy predicates that virus reactivation in latently infected cells is required to eliminate the human immunodeficiency virus (HIV) reservoir. In a recent study, we showed robust and persistent induction of plasma viremia in antiretroviral therapy (ART)-treated simian immunodeficiency virus-infected rhesus macaques (RMs) undergoing CD8α depletion and treated with the interleukin-15 (IL-15) superagonist N-803 (J. B. McBrien et al., Nature 578:154–159, 2020, https://doi.org/10.1038/s41586-020-1946-0). Of note, in that study we used an antibody targeting CD8α, thereby depleting NK cells, NKT cells, and γδ T cells, in addition to CD8^+^ T cells. In the current proof-of-concept study, we tested whether virus reactivation can be induced by administration of N-803 to simian-human chimeric immunodeficiency virus-infected, ART-treated RMs that are selectively depleted of CD8^+^ T cells via the CD8β-targeting antibody CD8b255R1. CD8β depletion was performed in five SHIV_SF162P3_-infected RMs treated with ART for 12 months and with plasma viremia consistently below 3 copies/ml. All animals received four weekly doses of N-803 starting at the time of CD8b255R1 administration. The induction of detectable plasma viremia was observed in three out of five RMs, with the level of virus reactivation seemingly correlated with the frequency of CD8^+^ T cells following CD8β depletion as well as the level of virus reactivation observed when the same animals underwent CD8α depletion and N-803 administration after 24 weeks of ART. These data indicate that CD8β depletion and N-803 administration can induce virus reactivation in SHIV_SF162P3_-infected RMs despite suboptimal depletion of CD8^+^ T cells and profound ART-induced suppression of virus replication, confirming a critical role for these cells in suppressing virus production and/or reactivation *in vivo* under ART.

**IMPORTANCE** The “shock and kill” HIV cure strategy attempts to reverse and eliminate the latent viral infection that prevents eradication of the virus. Latency-reversing agents tested in clinical trials to date have failed to affect the HIV viral reservoir. IL-15 superagonist N-803, currently involved in a clinical trial for HIV cure, was recently shown by our laboratory to induce robust and persistent induction of plasma viremia during ART in three *in vivo* animal models of HIV infection. These results suggest a substantial role for CD8^+^ lymphocytes in suppressing the latency reversal effect of N-803 by promoting the maintenance of viral latency. In this study, we tested whether the use of a CD8β-targeting antibody, which would specifically deplete CD8^+^ T cells, would yield similar levels of virus reactivation. We observed the induction of plasma viremia, which correlated with the efficacy of the CD8 depletion strategy.

## Abstract (precis)

The “shock and kill” HIV cure strategy attempts to reverse and eliminate the latent viral infection that prevents eradication of the virus. Latency-reversing agents tested in clinical trials to date have failed to affect the HIV viral reservoir. IL-15 superagonist N-803, currently involved in a clinical trial for HIV cure, was recently shown by our laboratory to induce robust and persistent induction of plasma viremia during ART in three *in vivo* animal models of HIV infection. These results suggest a substantial role for CD8^+^ lymphocytes in suppressing the latency reversal effect of N-803 by promoting the maintenance of viral latency. In this study, we tested whether the use of a CD8β-targeting antibody, which would specifically deplete CD8^+^ T cells, would yield similar levels of virus reactivation. We observed the induction of plasma viremia, which correlated with the efficacy of the CD8 depletion strategy.

## INTRODUCTION

Human immunodeficiency virus (HIV) is the causative agent of AIDS and infects approximately 36.7 million people worldwide (1). UNAIDS estimates there are 1.8 million new HIV infections annually and 1 million AIDS-related deaths (2). While antiretroviral therapy (ART) has dramatically reduced the mortality and morbidity of HIV infection, this treatment does not provide a cure for the infection.

The main obstacle to cure HIV infection is the presence of a population of long-lived, latently infected cells that harbors replication-competent virus and persists despite long-term ART (3–6). This reservoir is comprised primarily of resting memory CD4^+^ T cells (3–10). Recent data suggest that the viral reservoir of HIV is established early during infection (11, 12) and is responsible for the viral rebound observed after ART interruption (13, 14). Therefore, additional interventional strategies are necessary to cure HIV, and novel therapies targeting the viral reservoir of HIV are of the utmost importance. Infection of rhesus macaques (RMs) with simian immunodeficiency virus (SIV) or simian-human chimeric immunodeficiency virus (SHIV) are the most widely used animal models to study the mechanisms by which the viral reservoir is established and maintained under ART and to test preclinical interventions aimed at eliminating, or at least reducing, the viral reservoir *in vivo* (15).

The “shock and kill” HIV cure strategy seeks to reverse HIV latency and reactivate the viral reservoir (shock) to achieve its elimination via immunotherapeutic approaches (kill). In this view, a latency-reversing agent administered to ART-treated, HIV-infected individuals may be capable of activating CD4^+^ T cells to shock the integrated virus out of latency, leading to HIV RNA transcription, production of viral protein, and release of viral particles, potentially causing direct death of the infected cell via virus-mediated cytopathic effect and/or indirect killing via recognition by the immune system (16). The kill therapeutic aim of this strategy seeks to harness the cytolytic response of the immune system to eliminate infected cells shocked out of a previously latent state (16), thereby reducing the size of the viral reservoir.

While various latency-reversing agents have been tested in clinical trials in HIV-infected, ART-treated individuals, none have been shown to elicit the shock required to robustly reactivate the virus reservoir, failing to provoke even a minor increase in plasma viremia (17–22). A current clinical trial is aimed at disrupting the HIV reservoir using a novel latency-reversing agent, interleukin-15 (IL-15) superagonist N-803 (ClinicalTrials registration no. NCT02191098). N-803 is a complex of a mutant IL-15 and a dimeric IL-15 receptor αSu/Fc fusion protein (23). The engineered structure is at least 25 times more biologically potent than IL-15, as it mimics transpresentation, and the IgG-Fc component confers improved *in vivo* safety and bioavailability (24, 25). A recent study by our laboratory showed that N-803 induces robust and persistent plasma viremia in CD8-depleted, ART-treated, SIV-infected rhesus macaques, CD8-depleted, ART-treated, HIV-infected humanized mice, and CD8-depleted, ART-treated, SHIV-infected rhesus macaques (26). A feature of this study was the use of MT807R1, an anti-CD8α antibody, as the reagent administered to achieve CD8^+^ T cell depletion. As CD8α is expressed on NK, NKT, and γδ T cells, as well as CD8^+^ T cells, this treatment results in the depletion of multiple cellular subtypes, potentially confounding the interpretation of the results with respect to the specific role of CD8^+^ T cells.

In this proof-of-concept study, we sought to determine whether N-803 is capable of shocking the viral reservoir in ART-treated, SHIV-infected macaques depleted of CD8^+^ T cells using an antibody targeting CD8β, which is specifically expressed on CD8^+^ T cells. To directly compare the efficacy of both antibodies, we performed this experiment on a small cohort of SHIV-infected rhesus macaques that previously were depleted of CD8^+^ T cells using the antibody targeting CD8α, the results of which were recently published (26). We observed the induction of detectable plasma viremia in three out of five RMs, with the level of virus reactivation seemingly correlated with the frequency of CD8^+^ T cells following CD8β depletion, as well as the level of virus reactivation observed when the same animals underwent CD8α depletion and N-803 administration. These data indicate that CD8β depletion and N-803 administration can induce virus reactivation in SHIV-infected RMs despite suboptimal depletion of CD8^+^ T cells and profound ART-induced suppression of virus replication, confirming a critical role for CD8^+^ T cells in suppressing virus production and/or reactivation *in vivo* under ART.

## RESULTS

### Study design.

**FIG 1:**
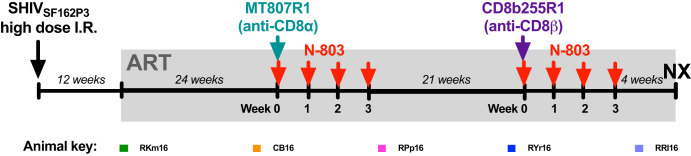
Study design. Animals were infected intrarectally with high-dose SHIV_SF162P3_, administered as a 1:50 dilution of a stock at 2,032 TCID_50_/ml, 109 RNA copies/ml, and 182 ng/ml P27. All animals were put on a three-drug ART regimen at 12 weeks post-SHIV infection consisting of tenofovir (TDF at 5.1 mg/kg/day or PMPA at 20 mg/kg/day), emtricitabine (FTC at 40 mg/kg/day), and dolutegravir (DTG at 2.5 mg/kg/day). ART was administered daily by subcutaneous injection for the remainder of the study. After 24 weeks of ART, animals were administered one dose of MT807R1 (anti-CD8α) at 50 mg/kg subcutaneously. N-803 was administered subcutaneously in a cycle of 100 μg/kg once a week for four consecutive weeks starting at the time of CD8α depletion. Twenty-four weeks after administration of MT807R1 with N-803, animals were administered one dose of CD8b255R1 (anti-CD8β) at 50 mg/kg subcutaneously. N-803 was administered subcutaneously in a cycle of 100 μg/kg once a week for four consecutive weeks starting at the time of CD8β depletion. An elective necropsy was performed 7 weeks after administration of CD8b255R1 with N-803. Abbreviations: I.R., intrarectal; ART, antiretroviral therapy; N-803, IL-15 superagonist complex; NX, necropsy.

Rhesus macaques were infected intrarectally with a high dose of SHIV_SF162P3_ and 12 weeks later were initiated on a daily three-drug regimen of ART (tenofovir, emtricitabine, and dolutegravir), which was maintained for the remainder of the study. To stabilize the latent viral reservoir, animals were treated with ART for 6 months prior to the first intervention, consisting of 50 mg/kg of body weight the anti-CD8α-depleting antibody MT807R1, with a cycle of N-803, administered at 100 μg/kg weekly for 4 weeks. Six months after the administration of MT807R1, following the reconstitution of CD8^+^ T cells, animals were administered 50 mg/kg the anti-CD8β-depleting antibody CD8b255R1, with an additional cycle of N-803 (Fig. 1). While sequential interventions allow comparison of the immunological effects of each intervention strategy, longer ART treatment at the time of CD8β depletion with N-803 prevents a direct virological comparison.

### N-803 administration induces virus reactivation in CD8β-depleted, ART-treated, SHIV-infected macaques.

**FIG 2:**
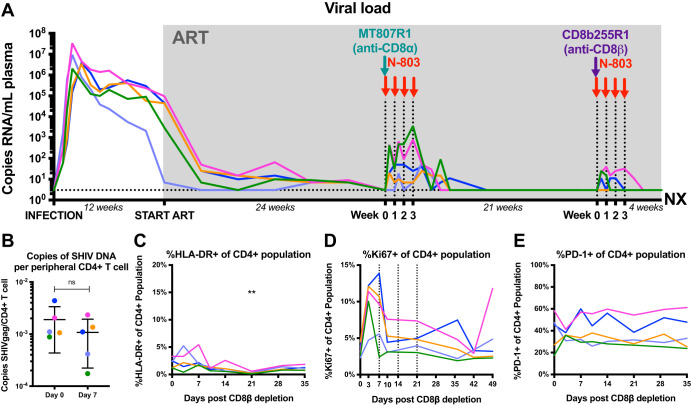
CD8β depletion with N-803 results in a modest virus reactivation correlated with CD8^+^ T cell frequencies but does not reduce the size of the SHIV reservoir. (A) Longitudinal plasma viral load starting at the time of infection, through ART initiation, CD8α depletion with N-803, CD8β depletion with N-803, and ending with animal necropsy. The limit of detection of the assay was 3 copies of SHIV RNA/ml of plasma. (B) Change in the level of cell-associated SHIV DNA in sorted, bulk, peripheral CD4^+^ T cells. Statistics were calculated using a Wilcoxon test. (C to E) Changes in the expression of HLA-DR (C), Ki67 (D), and PD-1 (E) on peripheral CD4^+^ T cells following intervention with CD8β depletion and N-803. Statistics were calculated using Friedman tests, and the level of significance for each comparison is indicated above the brackets (***, *P* < 0.001; **, *P* < 0.01; *, *P* < 0.05; nonsignificant [ns], *P* > 0.05). Each color designates a specific animal. Abbreviations: ART, antiretroviral therapy; NX, necropsy.

As previously published, N-803 resulted in robust and persistent virus reactivation in CD8α-depleted, ART-treated, SIV_mac239_-infected macaques, and these results were recapitulated, albeit to a lesser degree, in an SHIV_SF162P3_ model of infection as well as in HIV-infected humanized mice (26). While SHIV_SF162P3_ is derived from an SIV_mac239_ backbone, replacement of SIV *tat*, *rev*, and *env* with a corresponding sequence from HIV_SF162_ renders the infection less pathogenic (27). Thus, it was not surprising that the level of virus reactivation following CD8α depletion with N-803 was smaller in SHIV-infected macaques than in SIV-infected macaques. In this study, animals were treated with ART for 1 year prior to intervention, and viral loads were consistently less than 3 copies/ml of plasma. Following CD8β depletion with N-803, viral loads became detectable in 3/5 animals (Fig. 2A). While virus reactivation was observed in this pilot study, the levels were less impressive than those observed after (i) the previous CD8α depletion and N-803 administration in the same group of SHIV-infected macaques and (ii) CD8α depletion and N-803 administration in ART-treated, SIV-infected macaques (26). It must be emphasized, however, that in this study, CD8β depletion was conducted after a longer period of virus suppression with ART (i.e., 18 versus 12 months). Of note, CD8β depletion with N-803 did not result in a decrease in the size of the viral reservoir (Fig. 2B) but was associated with a transient increase in peripheral CD4^+^ T cell activation and proliferation (Fig. 2C to E).

### The anti-CD8β antibody was less effective than the anti-CD8α antibody in depleting CD8^+^ T cells.

**FIG 3:**
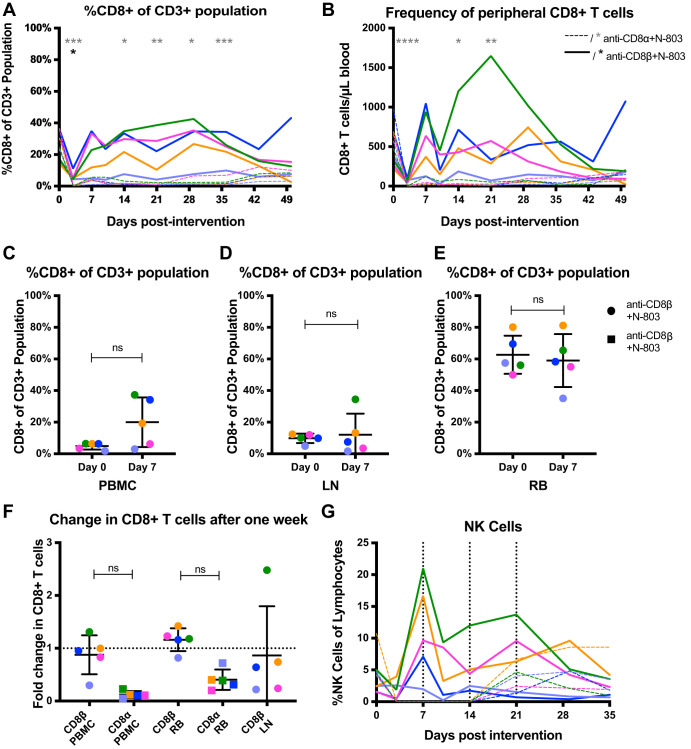
Reduction of CD8^+^ T cells following anti-CD8α or anti-CD8β. (A) Percentage of peripheral CD8^+^ cells from the CD3^+^ T cell population at the time of intervention (day 0) through day 49. (B) Longitudinal frequency of peripheral CD8^+^ T cells. (C to E) Percentage of CD8^+^ cells from the CD3^+^ T cell population at the start of intervention and after 7 days in the peripheral blood (C), lymph node (via a fine-needle aspirate) (D), and the rectum (via a rectal biopsy specimen) (E). (F) Fold change of CD8^+^ T cells 7 days after the start of intervention in the peripheral blood and rectum with CD8β depletion and CD8α depletion. This was calculated by dividing the percentage of CD8^+^ T cells at day 0 by that at day 7. Of note, fine needle aspirates of the lymph node were not collected following CD8α depletion. (G) The number of NK cells (CD3^−^ CD20^−^ CD14^−^ HLA-DR^−^ NKG2A^+^) present in peripheral blood longitudinally following the start of interventions. Intervention with anti-CD8β and N-803 is shown with solid lines or circles, and asterisks denoting statistical significance are black. Intervention with anti-CD8α is shown with dashed lines or squares, and asterisks denoting statistical significance are gray. Statistics for panels A, B, and G were calculated using Friedman tests and for panels C to F were calculated using two-sided Wilcoxon tests. The level of significance for each comparison is indicated above the brackets (****, *P* < 0.0001; ***, *P* < 0.001; **, *P* < 0.01; *; *P* < 0.05; nonsignificant, *P* > 0.05). Each color represents a specific animal. Abbreviations: PBMC, peripheral blood mononuclear cells; LN, lymph node; RB, rectal biopsy specimen; ns, not significant.

CD8α is expressed on CD8^+^ T cells at a very high density, either as a homodimer or a heterodimer with CD8β, while CD8β is expressed at a lower density and always in a heterodimeric form with CD8α (28). Thus, while both MT807R1 and CD8b255R1 antibodies are of the IgG1 isotype, they target epitopes with various levels of availability, which may affect their ability to deplete CD8^+^ T cells. In fact, previous studies utilizing CD8b255R1 as a CD8^+^ T cell depletion strategy have observed suboptimal CD8^+^ T cell depletion in rhesus macaques (29, 30). In this study, we next evaluated the ability of CD8b255R1 to deplete CD8^+^ T cells *vis-à-vis* the level of depletion achieved with MT807R1. As shown in Fig. 3A and B, the percentage and frequency of peripheral CD8^+^ T cells reached a brief nadir early after CD8β depletion. In contrast, CD8α depletion resulted in a sustained decrease in CD8^+^ T cells. Additionally, the percentage of CD8^+^ T cells was not significantly different in the periphery, lymph node (LN), and rectum 1 week after CD8β depletion with N-803 (Fig. 3C to E). While some animals saw an increase in the percentage of CD8^+^ T cells across tissues following CD8β depletion with N-803, the fold change was not significantly different from that following CD8α depletion with N-803 (Fig. 3F). We also calculated the frequency of circulating NK cells following both interventions (Fig. 3G). While CD8α depletion with N-803 resulted in a transient depletion of NK cells, as predicted, CD8β depletion with N-803 did not deplete the NK cell population.

### The level of virus reactivation after CD8^+^ T cell depletion is inversely correlated with the residual CD8^+^ T cell counts.

**FIG 4:**
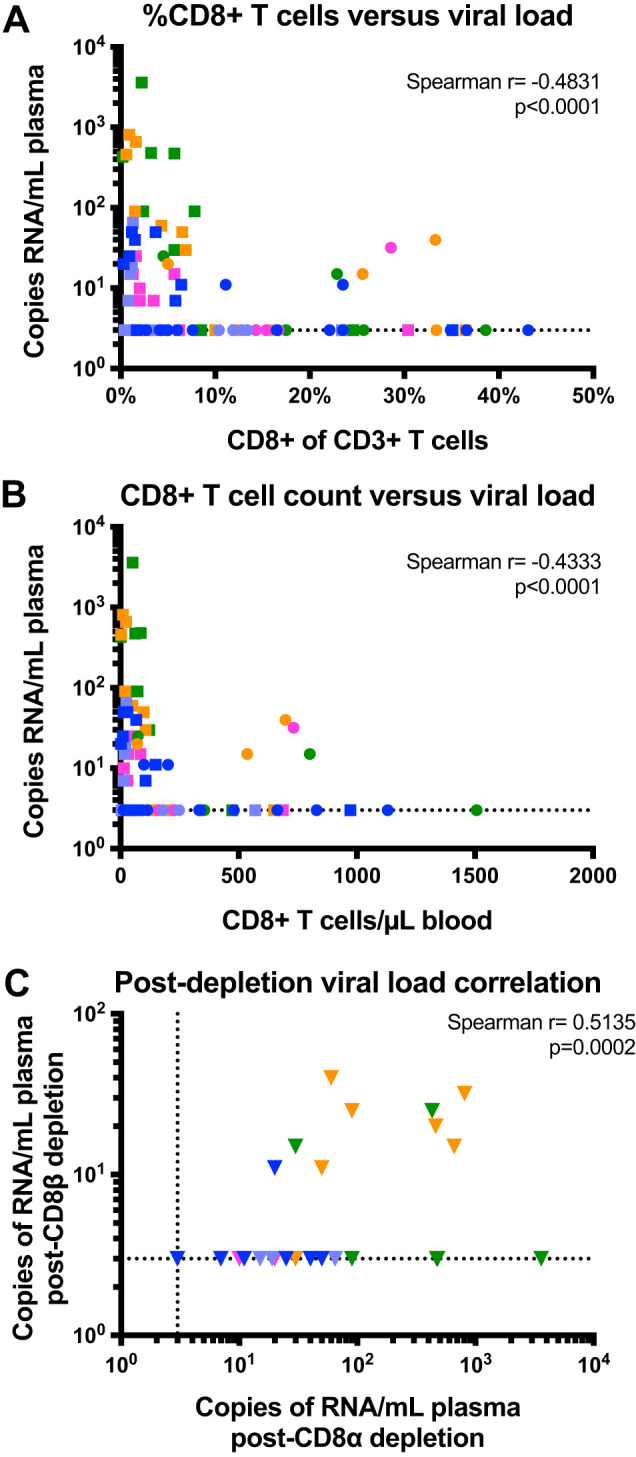
Level of virus reactivation is correlated with the efficacy of CD8^+^ T cell depletion and previous depletions. (A and B) Correlation between the percentage of peripheral CD8^+^ T cells and plasma viral load (A) and peripheral CD8^+^ T cell count and viral load (B). Counts and viral loads from both depletion strategies were combined for the correlations. (C) Correlation between plasma viral loads following CD8α depletion and CD8β depletion. Correlations were calculated via Spearman’s rho. Each color designates a specific animal. Circles represent intervention using CD8β depletion with N-803, and squares represent intervention using CD8α depletion with N-803.

A previous study by our group indicated that the level of virus reactivation following N-803 in CD8-depleted, ART-treated, SIV-infected rhesus macaques is inversely correlated with postdepletion CD8^+^ T cell frequency (26). Therefore, we hypothesized that the level of virus reaction following CD8 depletion and N-803 administration would be dependent on the ability of the particular anti-CD8 antibody to deplete CD8^+^ T cells. As shown in Fig. 4A and B, we found very strong negative correlations between both percentage and absolute count of CD8^+^ T cells in the periphery and the plasma viral load, suggesting that the more modest virus reactivation observed following CD8β depletion with N-803 is due in part to suboptimal CD8^+^ T cell depletion. Additionally, we sought to determine whether there was a correlation between postdepletion viral loads observed following CD8α and CD8β depletion, and we found that the level of virus reactivation was correlated between sequential depletions (Fig. 4C).

## DISCUSSION

The shock-and-kill approach to curing HIV infection relies on the reactivation of latent virus followed by immune-mediated clearance of cells that have reactivated virus production as a strategy to reduce and ultimately eliminate the virus reservoir that persists under ART. A recent study by our group showed that the combination of the IL-15 superagonist N-803 and CD8^+^ lymphocyte depletion induced a robust and persistent induction of plasma viremia in ART-treated, SIV-infected macaques and HIV-infected humanized mice (26). These results are compatible with a substantial *in vivo* role for CD8^+^ lymphocytes in suppressing the latency reversal effect of N-803 and, therefore, in promoting the maintenance of viral latency under ART. A key feature of these models was the use of a depleting antibody targeting CD8α, which is expressed not only on CD8^+^ T cells but also on NK cells, NKT cells, and γδ T cells. As such, the relative contribution of these cell types in mediating the *in vivo* suppression of SIV and HIV transcription under ART remains unknown. In this proof-of-concept study, we tested whether virus reactivation follows the administration of N-803 to SHIV-infected, ART-treated macaques that underwent selective depletion of CD8^+^ T cells using a CD8β-targeting antibody. To specifically compare the efficacy of these two depletion strategies, we performed the combined intervention with N-803 and anti-CD8β depletion in five SHIV-infected, ART-treated macaques that were previously treated with N-803 and anti-CD8α antibodies (26).

Importantly, CD8β depletion with N-803 resulted in measurable virus reactivation in three out of five ART-treated, SHIV-infected macaques. As expected, given the lower levels of viremia of SHIV-infected macaques compared to those of SIV-infected ones, we observed lower levels of virus reactivation than those in our previous study using an SIV model of infection (26). In addition, we found lower levels of virus reactivation after CD8β depletion versus CD8α depletion. This finding is also not surprising, given that MT807R1 induces a more profound and persistent CD8 depletion in both peripheral blood and tissues than CD8b255R1. Indeed, CD8^+^ T cells were no longer significantly depleted across tissues 1 week after administration of CD8b255R1 with N-803. As such, multiple administrations of CD8b255R1 may be necessary to achieve more effective and sustained CD8^+^ T cell depletion. It is also important to note that the experimental design of this pilot study involved the use of CD8b255R1 after a more prolonged period of ART-mediated suppression of viremia (i.e., 18 months versus 12 months), a setting of likely smaller reservoir size and increased reservoir stability that may have hampered the effect of the combined CD8b255R1 and N-803 administration. Finally, we cannot formally rule out that in the current study the effect of N-803 at the time of CD8β depletion was somewhat limited by anti-N803 antibodies produced after the first cycle of administration of this molecule (i.e., at the time of CD8α depletion). However, the observation of a clear biological effect of N-803 in terms of NK and CD4^+^ T cell proliferation makes this possibility unlikely.

All these limitations notwithstanding, the current study is useful in that it makes two points relatively clearly. The first is that a selective CD8^+^ T cell depletion that does not involve other cell types (such as CD8α^+^ NK cells) is still able to reveal the latency-reversing activity of N-803. This points to a direct role of CD8^+^ T cells, which is also supported by the observation that the level of virus reactivation was inversely correlated with the frequency of residual CD8^+^ T cells following CD8β depletion. Further, studies of CD8α and CD8β depletion, in which both interventions achieve the same level of CD8^+^ T cell depletion in both blood and tissues, will be required to dissect whether and to what extent the concomitant depletion of NK cells caused by the CD8α-depleting MT807R1 antibody contributes to the latency-promoting activity of CD8^+^ T cells. The second point is that even in a setting of prolonged ART suppression of virus replication (i.e., 18 months), incomplete and transient CD8^+^ T cell depletion, and use of SHIV_SF162P3_ that causes lower levels of viremia than SIV, the combination of CD8^+^ T cell depletion and N-803 administration was sufficient to induce detectable reactivation of virus production, further confirming and expanding previous observations from our team (26, 31).

A recent small study by the group of Okoye that was publicly presented but is not yet published (32) failed to observe virus reactivation in 4 out of 5 MaMuB*08-negative and MaMuB*17-negative, ART-treated, SIV-infected macaques that underwent CD8β depletion. In this study, however, ART was initiated within 2 weeks from the initial SIV infection (as opposed to the 8 to 12 weeks of our studies), creating an experimental setting in which (i) the reservoir size is likely smaller than that in our experiments and (ii) CD8^+^ T cells have been exposed to SIV for a much shorter time. In addition, the overall level of CD8^+^ T cell depletion achieved in the Okoye study was variable and not always very profound (31). For these reasons, we believe that these limited observations, while certainly interesting, are of no particular relevance to our large set of studies that revealed a strong and robust effect of CD8^+^ lymphocyte depletion in directly inducing (when used alone) and/or indirectly promoting (when used in combination with N-803) virus reactivation under ART in SIV- and SHIV-infected macaques.

In conclusion, this pilot study indicates that CD8β depletion and N-803 administration can induce virus reactivation in SHIV_SF162P3_-infected RMs despite suboptimal depletion of CD8^+^ T cells and profound ART-induced suppression of virus replication, confirming a critical role for these cells in suppressing virus production *in vivo* under ART. Future studies of shock-and-kill cure strategies in SIV- or SHIV-infected macaques could incorporate CD8β depletion using the CD8b255R1 antibody to achieve virus reactivation.

## MATERIALS AND METHODS

### Study approval.

All animal experiments were conducted by following the guidelines established by the Animal Welfare Act and the *Guide for the Care and Use of Laboratory Animals* (33) and were performed in accordance with institutional regulations after review and approval by the Institutional Animal Care and Usage Committee (IACUC; 3000065, 2003297, 2003470, and PROTO201700665) at the Yerkes National Primate Research Center (YNPRC; Atlanta, GA). Anesthesia was administered prior to performing any procedure, and the proper steps were taken to minimize any suffering the animals may have experienced.

### Animals, SIV infection, and antiretroviral therapy.

This study was conducted using a total of five Indian-origin rhesus macaques housed at Yerkes National Primate Research Center. All RMs were HLA*B07^−^ and HLA*B17^−^. All procedures were approved by the Emory University IACUC, and animal care facilities are accredited by the U.S. Department of Agriculture (USDA) and the Association for Assessment and Accreditation of Laboratory Animal Care (AAALAC) International.

Animals were infected intrarectally with high-dose SHIV_SF162P3_, administered as a 1:50 dilution of a stock of 2,032 50% tissue culture infective doses (TCID_50_)/ml, 10^9^ RNA copies/ml, and 182 ng/ml P27. Three of 5 animals were administered broadly neutralizing antibody targeting V3-glycan PGT-121 (RPp16, 1 mg/kg; RRl16 and RYr16, 0.2 mg/kg) at the time of infection as part of a previous study. The presence of the antibody did not prevent infection.

All animals were put on a three-drug ART regimen at 12 weeks post-SHIV infection, after the washout period of PGT-121 had passed. Tenofovir (TDF at 5.1 mg/kg/day or PMPA at 20 mg/kg/day) and emtricitabine (FTC at 40 mg/kg/day) both were kindly provided by Gilead Pharmaceuticals. Dolutegravir (DTG at 2.5 mg/kg/day) was kindly provided by ViiV Pharmaceuticals. Drugs were administered daily by subcutaneous injection.

### CD8 depletions and N-803 administration.

After 6 months of ART, animals were administered one dose of the anti-CD8α-depleting antibody MT-807R1 at 50 mg/kg subcutaneously. N-803 was administered subcutaneously in a cycle of 100 μg/kg once a week for four consecutive weeks starting at the time of CD8 depletion. Six months after CD8α depletion, animals were administered one dose of the anti-CD8β-depleting antibody CD8b255R1 at 50 mg/kg subcutaneously. N-803 again was administered subcutaneously in a cycle of 100 μg/kg once a week for four consecutive weeks, starting at the time of CD8 depletion.

### Sample collection and tissue processing.

Blood was collected longitudinally throughout the study. Fine-needle aspirates of the lymph node (LN) and rectal tissue (rectal biopsy specimen) were collected prior to intervention and 1 week after intervention and processed for further analyses, as previously described (30).

### Immunophenotype by flow cytometry.

Multiparametric flow cytometry was performed according to a standard protocol on peripheral blood mononuclear cells (PBMC), cells collected from LN fine-needle aspirates, and rectal cells using fluorescently labeled monoclonal antibodies cross-reactive in RM. In addition to the viability dye LIVE/DEAD fixable aqua, the following antibodies were used at room temperature for 30 min: CD3 allophycocyanin-Cy7 (SP34-2), CD4 BV650 (OKT4), CD8α BV711 (RPA-T8), CD8β phycoerythrin (PE)-Cy5 (SIDI8BEE), CD28 ECD (CD28.2), HLA-DR peridinin chlorophyll protein-Cy5.5 (G46-6), PD-1 BV421 (EH12.2H7), Ki67 AF700 (B56), CD20 PE-Cy5 (2H7), CD14 PE-Cy7 (M5E2), and NKG2A (CD159) PE (Z199). Because of epitope masking by the depleting antibody, CD8α was used to identify CD8^+^ T cells following CD8β depletion and CD8β was used to identify CD8^+^ T cells following CD8α depletion.

All flow cytometry specimens were acquired on an LSR II (BD Biosciences) equipped with fluorescence-activated cell sorter software (FACS Diva), and analyses of the acquired data were performed using FlowJo software (Tree Star).

### Determination of plasma SHIV RNA and cell-associated SHIV *gag* DNA.

Plasma SHIV *gag* RNA was examined using quantitative PCR to determine SHIV plasma viral load essentially as described previously, using high-sensitivity assay formats (34, 35). CD4^+^ T cells were sorted from PBMC via CD4 MicroBeads (Miltenyi 130-091-102), purified CD4^+^ T cells were preserved as a dry pellet, and the quantification of total cell-associated *gag* DNA was performed as previously described (36).
